# Transcriptomes of three species of Tipuloidea (Diptera, Tipulomorpha) and implications for phylogeny of Tipulomorpha

**DOI:** 10.1371/journal.pone.0173207

**Published:** 2017-03-06

**Authors:** Zehui Kang, Xiao Zhang, Shuangmei Ding, Chufei Tang, Yuyu Wang, Herman de Jong, Stephen L. Cameron, Mengqing Wang, Ding Yang

**Affiliations:** 1 Department of Entomology, China Agricultural University, Beijing, China; 2 Naturalis Biodiversity Center, Darwinweg, CR Leiden, the Netherlands; 3 Department of Entomology, Purdue University, West Lafayette, Indiana, United States of America; 4 Institute of Plant Protection, Chinese Academy of Agricultural Sciences, Beijing, China; Beijing Institute of Genomics Chinese Academy of Sciences, CHINA

## Abstract

Tipulomorpha has long been a problematic taxon in terms of familial composition, phylogenetic relationships among families and position relative to other ‘lower’ Diptera. Whole-transcriptome shotgun sequencing provides a powerful basis for phylogenetic studies. We performed *de novo* transcriptome sequencing to produce the first transcriptome datasets representing the families Pediciidae, Limoniidae and Cylindrotomidae using high-throughput sequencing technologies. We assembled cDNA libraries for *Pedicia vetusta* (Alexander) (Pediciidae), *Rhipidia sejuga* Zhang, Li and Yang (Limoniidae) and *Liogma simplicicornis* Alexander (Cylindrotomidae). Using the Illumina RNA-Seq method, we obtained 28,252, 44,152 and 44,281 unigenes, from the three respective species. Based on sequence similarity searches, 12,475 (44.16%), 20,334 (46.05%) and 17,478 (39.47%) genes were identified. Analysis of genes highly conserved at the amino acid sequence level revealed there were 1,709 single-copy orthologs genes across the analyzed species. Phylogenetic trees constructed using maximum likelihood (ML) based on the 1,709 single-copy orthologs genes indicated that the relationship between the four major infraorders of lower Diptera was: Culicomorpha + (Tipulomorpha + (Psychodomorpha + (Bibionomorpha + Brachycera))). Trichoceridae belongs within Tipulomorpha as the sister-group of Tipuloidea. Highly supported relationships within the Tipuloidea are Pediciidae + (Limoniidae + (Cylindrotomidae + Tipulidae)). Four-cluster likelihood mapping was used to study potential incongruent signals supporting other topologies, however, results were congruent with the ML tree.

## Introduction

**Fig 1:**
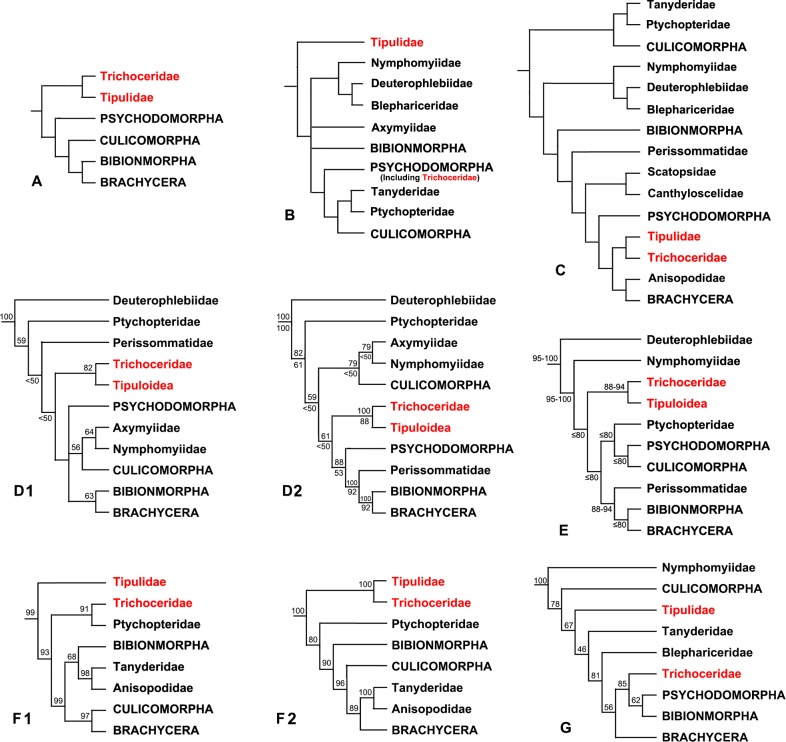
Phylogenetic hypotheses of lower Diptera relationships from previous analyses. **(A)** Hennig [4]. Phylogenetic hypothesis of lower Diptera relationships based primarily on imaginal characters. **(B)** Wood & Borkent [14]. Cladogram showing relationships between the families of the Nematocera. **(C)** Oosterbroek & Courtney [9]. Cladogram of the families of nematocerous Diptera. **(D)** Bertone *et al*. [10]. 1) Parsimony analysis of combined nuclear ribosomal (28S) and protein-coding (CAD, PGD and TPI) genes (bootstrap values (BV) shown above branches). 2) Majority rule consensus of Bayesian Markov chain Monte Carlo (posterior probabilities (PP) shown above branches and bootstrap values shown below branches). **(E)** Wiegmann *et al*. [16]. Combined molecular phylogenetic tree for Diptera (BV shown above branches shown above or below branches). **(F)** Beckenbach [17]. 1) Mitochondrial phylogenetic tree of major groups of Diptera derived from a Bayesian analysis of all major mitochondrial protein coding genes (PP shown above branches). 2) Bayesian mitochondrial tree using codon positions 1 and 2 for cox1–3, cytb, and atp6 genes, and all alignable sites for the ribosomal genes (PP shown above branches). **(G)** Lambkin *et al*. [18]. The Bayes combined majority rule consensus tree (PP shown above branches).

The infraorder Tipulomorpha is one of the most speciose groups of flies [1–2]. According to Hennig, this infraorder included four families, Trichoceridae, Tipulidae, Cylindrotomidae and Limoniidae, of which Trichoceridae was considered the sister-group of the remaining Tipulomorpha [3–5]. This arrangement of Tipulomorpha, containing both Trichoceridae and Tipuloidea (= Tipulidae *sensu lato*, or Cylindrotomidae, Limoniidae, Pediciidae, and Tipulidae sensu stricto) was accepted by Dahl [6], Griffiths [7], Starý [8], Oosterbroek & Courtney [9] and Bertone *et al*. [10]. Hennig further hypothesized that Tipulomorpha was the sister-group of all remaining Diptera, a classification accepted by Krzeminski [11], Michelsen [12] and Blagoderov *et al*. [13], and partly accepted by Wood & Borkent [14]. Based on larval characters, Wood & Borkent considered that the concept of Tipulomorpha was restricted to just Tipuloidea, and the infraorder was sister-group to all other Diptera, while Trichoceridae was assigned to the Psychodomorpha [14]. This shift of Trichoceridae from the Tipulomorpha to nested within Psychodomorpha was also suggested by Friedrich & Tautz [15]. Although Oosterbroek & Courtney supported the sister-group relationship between Trichoceridae and Tipuloidea, they considered that Tipulomorpha was the sister-group of the clade Anisopodidae + Brachycera, and as such were a group of ‘higher’ Nematocera [9], a much more derived position than conceived by Hennig and followers [3–5]. More recent molecular and morphological studies have examined the higher classification of Diptera [10,16–18], however, there is still no widely accepted hypothesis of the relationships among lower dipteran families, including the relationship among the four major nematoceran infraorders (Bibionomorpha, Culicomorpha, Psychodomorpha and Tipulomorpha). Tipulomorpha was found as the earliest branching of the four major infraorders of lower Diptera by Wiegmann *et al*. [16] and Beckenbach [17], while Lambkin *et al*. suggested Culicomorpha as the earliest branching infraorder [18]. Both the views were supported by Bertone *et al*. when using different analysis methods [10] (Fig 1).

Interfamilial relationships in the Tipuloidea are also unresolved. Tipuloidea was treated as a single family by Alexander [19–20], Savchenko [21–23] and Brodo [24]. The alternative classification as Tipuloidea with four families was used and supported by Hennig [4], Oosterbroek & Theowald [25] and Oosterbroek [26]. However, in both classification schemes, Pediciidae was recovered as nested within Limoniidae by both sets of studies. Alexander [19–20] and Savchenko [21–23] presented the earliest evolutionary hypotheses of Tipuloidea, which were qualitative and recovered relationships based on unstated criteria. Both of them considered Tipulidae as the sister-group of the remaining Tipuloidea. Starý raised the subfamily Pediciinae to full family rank and constructed a phylogenetic tree for Tipulomorpha, finding a monophyletic Limoniidae to be the sister-group to a clade containing Pediciidae + (Cylindrotomidae + Tipulidae) [8]. Recent studies revealed new insights into the higher-level classification of Tipuloidea and have suggested that, rather than Limoniidae, Pediciidae was the sister-group to the remaining Tipuloidea [27–28].

Whole-transcriptome shotgun sequencing provides a powerful basis for phylogenetic studies, and provides a means to overcome the limitations of multi-locus PCR based molecular phylogenetics [29–30]. In this study, we sequenced the cDNA from three species, *P*. *vetusta*, *R*. *sejuga* and *L*. *simplicicornis*, belonging respectively to the Pediciidae, Limoniidae and Cylindrotomidae. Additionally, we analyzed published transcriptome sequence data and annotated gene sets from publically available draft genome sequences. Based on data from representatives of the Trichoceridae, four families of Tipuloidea and the other three infraorders, we constructed a phylogenetic tree using maximum likelihood (ML). In addition, we applied Four-cluster Likelihood Mapping (FcLM) to study potential incongruent signal, which might not be revealed by traditional phylogenetic methods.

## Materials and methods

### Ethics statement

No specific permits were required for the specimens collected for this study. The specimens were common in China and the field studies did not involve endangered or protected species. The species were not included in the “List of Protected Animals in China”.

### Specimen collection, preservation and RNA extraction

The specimens used for the RNA extraction and sequencing transcriptome were collected from several different regions of China. Specimens were immersed alive in RNAlater and crushed with sterile forceps immediately upon wild collection. Then they were stored at -80°C until further processing. The number of specimens, stage, sex details, preserved information and collection data are listed in S1 Table.

For each species, total RNA was extracted using the Trizol reagent according to the manufacturer’s instructions (Invitrogen, CA, USA). RNA contamination and degradation was monitored on 1% agarose gels. Other quality parameters, such as purity, concentration and integrity, were examined using the NanoPhotometer® spectrophotometer (IMPLEN, CA, USA), the Qubit® RNA Assay Kit run on the Qubit®2.0 Flurometer (Life Technologies, CA, USA), and the RNA Nano 6000 Assay Kit run on the Agilent Bioanalyzer 2100 system (Agilent Technologies, CA, USA).

### Library preparation and transcriptome sequencing

Three cDNA libraries were prepared employing NEBNext®Ultra™ RNA Library Prep Kit for Illumina® (NEB, USA), in each case using 3μg of total RNA. Messenger RNA was isolated by Poly-T oligo-attached magnetic beads and fragmented in fragmentation buffer under elevated temperature. After the first strand cDNA was synthesized, the second-strand cDNA synthesis was performed using DNA Polymerase I and RNase H. Libraries were then size-selected for cDNA target fragments of 150–200 bp with 3 μl of USER Enzyme (NEB, USA); this was followed by PCR amplification using Phusion High-Fidelity DNA polymerase, Universal PCR primers and Index (X) Primer. PCR products were purified using the AMPure XP system. The library preparations were sequenced using the Illumina HiSeqTM 2000 system.

### Transcriptome assembly

Raw data were filtered to remove low quality reads, and reads containing adapter or Poly-N sequences. Quality parameters of clean data, such as Q20, Q30, GC-content and sequence duplication level, were calculated. Transcriptome assembly was accomplished using Trinity [31] with min_kmer_cov set to 2 and all other parameters set default based on the left.fq and right.fq pooled by the left files (read1 files) and right files (read2 files) for each library. The assembly process implemented in Trinity can be divided into three main steps. Firstly, all reads were broken into defined K-mers. Subsequently, these k-mers were merged to form edges, which were reported as contigs and then the contigs were clustered into components for construction of de Bruijn graphs. Lastly, real reads were used to resolve ties in the de Bruijn graphs and generate transcript sequences.

### Published data and orthology assignment

Nucleotide sequence assemblies of published transcriptome data were obtained from the NCBI's Transcriptome Sequences Database (TSA) and other various web sources (S2 Table). We predicted the CDS and amino acid sequences using TransDecoder v1.0.0 (available on Github https://github.com/TransDecoder/TransDecoder).

The ortholog reference set for orthology assignment was selected on the basis of the database OrthoDB7 (http://cegg.unige.ch/orthodb7 and http://cegg.unige.ch/orthodb/browse). We compiled a set of genes that are single-copy orthologs among Diptera and occur in the genomes of each of the following eight reference species: *Aedes aegypti*, *Anopheles gambiae*, *Culex quinquefasciatus*, *Lutzomyia longipalpis*, *Phlebotomus papatasi*, *Mayetiola destructor*, *Drosophila melanogaster* and *Drosophila persimilis*. OrthoDB 7 specified 1,709 single-copy protein-coding genes (S3 Table). We downloaded the amino acid sequences that are associated with each of the 1,709 single-copy ortholog groups (OGs) along with their available gene description (S4 Table). We aligned the amino acid sequences for each OG with MAFFT v7.205 [32–33] using the L-INS-i alignment algorithm and then build pHMMs with the program hmmbuild from the HMMER 3.0 software package [34] on the basis of the resulting multiple amino acid sequence alignments.

Orthology assignment of the transcriptomes was assessed by HaMStR v13.2.4 [35]. We ran HaMStR with the following settings: (i) the E-value cut-off for the pHMM search was 1e-5, (ii) the reciprocity criterion was considered fulfilled if the candidate OG was found as best hit in at least one of the 8 reference species during the reciprocal best hit search (RBH) (relaxed option), (iii) in case of multiple transcripts being assigned to a given OG, the best set of non-overlapping transcripts was chosen while non- overlapping transcripts are automatically concatenated (representative option). We searched and removed all multiple-assigned transcripts since it was possible that a given transcript was assigned to more than one OG.

### Phylogenetic and FcLM analysis

Phylogenetic analysis was conducted on the alignments obtained using MAFFT v7.205 [32–33]. Spurious sequences or poorly aligned regions were removed from the multiple sequence alignment using trimAl v1.2 (automated1 option). ML analyses were constructed using PhyML v 3.0 with the default choice JTT [36]. The tree searching algorithm used the SPR options provided by PhyML. In order to analyze single phylogenetic splits, FcLM analyses were completed using the program TreePuzzle v5.3 [37–38]. We selected the following two phylogenetic hypotheses concerning the Tipulomorpha for FcLM testing: 1) which infraorder is the sister-group of the remaining Diptera, Culicomorpha or Tipulomorpha; 2) is Trichoceridae part of Tipulomorpha, or Psychodomorpha as suggested by Wood & Borkent [14]. For each hypothesis, all species in the decisive dataset were grouped into four clusters representing alternative resolutions of the phylogenetic question of interest.

### Data deposition

The transcriptome sequencing data have been deposited in the NCBI Sequence Read Archive (SRA) database under the following accession numbers: SRR3452301 (*P*. *vetusta*), SRR3452300 (*R*. *sejuga*) and SRR3441821 (*L*. *simplicicornis*). The assembled transcriptomes have been deposited in the NCBI Transcriptome Shotgun Assembly (TSA) database under the accession numbers GEMI00000000, GEMJ00000000 and GEMK00000000 respectively.

## Results

### Transcriptome sequencing and assembly

**Fig 2:**
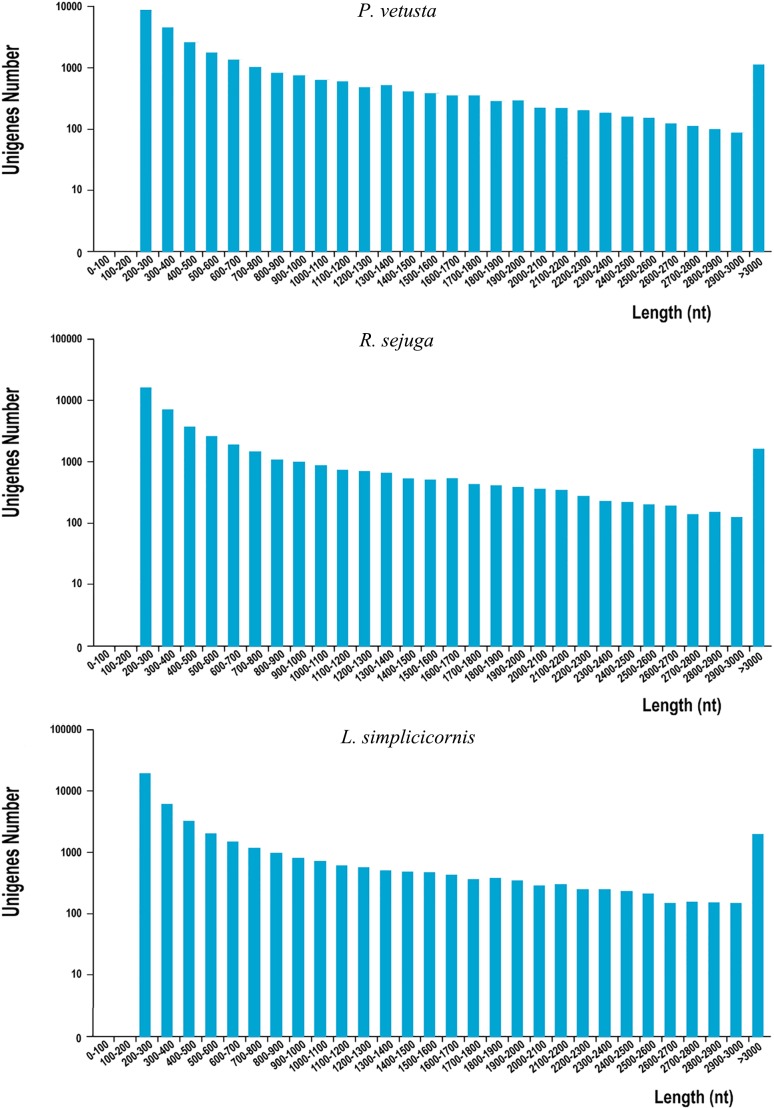
Sequence-length distribution of unigenes. The X-axis represents the length range bins; the Y-axis is the amount of transcripts present.

In this study, the transcriptomes were sequenced using the Illumina sequencing method. In total, 23,026,828 (Q30 96.69%), 24,843,179 (Q30 96.66%)and 28,349,912 (Q30 93.10%) clean reads were generated for *P*. *vetusta*, *R*. *sejuga* and *L*. *simplicicornis* respectively. We obtained 1,003,377, 2,110,937 and 2,964,958 contigs from the three species. A total of 41,870, 60,530 and 71,569 transcripts were confirmed. In *P*. *vetusta*, 28,252 unigenes were detected with a total length of 23,378,916 bp. The N50 and average length was 1,458 bp and 828 bp, respectively. In *R*. *sejuga*, 44,152 unigenes were obtained with an average length of 770 bp and a N50 length of 1,403 bp. Those unigenes comprised 34,004,882 bp totally. In *L*. *simplicicornis*, we found 44,281 unigenes. Those unigenes comprised 33,946,199 bp totally, with an average length of 767 bp and a N50 length of 1602 bp (Table 1). The sequence length distributions of unigenes for the three species are indicated in Fig 2.

**Table 1:** Length distribution of the contigs, transcripts and unigenes clustered from the *De novo* assembly.

Species	Length Range	Contig	Transcript	Unigene
*P*. *vetusta*	200–300	982,473 (97.92%)	9,711 (23.19%)	8,736 (30.92%)
	300–500	7,594 (0.76%)	8,607 (20.56%)	7,014 (24.83%)
	500–1,000	6,009 (0.60%)	8,213 (19.62%)	5,608 (19.85%)
	1,000–2,000	4,517 (0.45%)	7,888 (18.84%)	4,240 (15.01%)
	2,000 +	2,784 (0.28%)	7,451 (17.80%)	2,654 (9.39%)
	Total Number	1,003,377	41,870	28,252
	Total Length	65,247,903	50,313,936	23,378,916
	N50 Length	78	2,234	1,458
	Average Length	65	1,202	828
*R*. *sejuga*	200–300	2,081,076 (98.59%)	18,266 (30.18%)	15,925 (36.07%)
	300–500	11,758 (0.56%)	13,463 (22.24%)	10,771 (24.40%)
	500–1,000	8,412 (0.40%)	11,348 (18.75%)	7,933 (17.97%)
	1,000–2,000	5,912 (0.28%)	9,744 (16.10%)	5,721 (12.96%)
	2,000 +	3,779 (0.18%)	7,709 (12.74%)	3,802 (8.61%)
	Total Number	2,110,937	60,530	44,152
	Total Length	127,371,529	59,895,908	34,004,882
	N50 Length	52	1,876	1,403
	Average Length	60	990	770
*L*. *simplicicornis*	200–300	2,937,000 (99.06%)	23,604 (32.98%)	19,399 (43.81%)
	300–500	11,153 (0.38%)	13,655 (19.08%)	9,448 (21.34%)
	500–1,000	7,417 (0.25%)	11,909 (16,64%)	6,468 (14.61%)
	1,000–2,000	5,132 (0.17%)	10,694 (14.94%)	4,858 (10.97%)
	2,000 +	4,256 (0.14%)	11,707 (16.36%)	4,108 (9.28%)
	Total Number	2,964,958	71,569	44,281
	Total Length	161,642,392	78,482,160	33,946,199
	N50 Length	49	2,290	1,602
	Average Length	55	1,097	767

### Functional annotation

The unigenes of the three species were annotated by searching against the NR [39], GO [40], COG [41], KOG, KEGG [42], Pfam [43], and Swiss-Prot databases (Table 2).

**Table 2:** The numbers and distribution rate of unigenes in the databases of NR, GO, COG, KOG, KEGG, Pfam and Swiss-Prot.

Database	Number (Percentage) of annotated unigenes		
	*P*. *vetusta*	*R*. *sejuga*	*L*. *simplicicornis*
NR	11,989 (42.44%)	19,345 (43.81%)	16,928 (38.23%)
GO	7,900 (27.96%)	11,005 (24.93%)	9,415 (21.26%)
COG	3,398 (12.03%)	7,170 (16.24%)	4,978 (11.24%)
KOG	8,993 (31.83%)	13,754 (31.15%)	10,973 (24.78%)
KEGG	6,091 (21.56%)	9,419 (21.33%)	5,999 (13.55%)
Pfam	9,094 (32.19%)	14,762 (33.43%)	11,441 (25.84%)
Swiss-Prot	7,396 (26.18%)	11,501 (26.05%)	9,369 (21.16%)
All	12,475 (44.16%)	20,334 (46.05%)	17,478 (39.47%)

Of the *P*. *vetusta* unigenes, 12,475 (44.16%) were found in at least one of the seven public databases,11989 (42.44%) had significant matches in the NR database, 7,900 (27.96%) in GO, 3,398 (12.03%) in COG, 8,993 (31.83%) in KOG, 6,091 (21.56%) in KEGG, 9,094 (32.19%) in Pfam and 7,396 (26.18%) in Swiss-Prot.

There were 20,334 unigenes (46.05%) from *R*. *sejuga* found in at least one database, 19,345 (43.81%) in NR, 11,005 (24.93%) in GO, 7,170 (16.24%) in COG, 13,754 (31.15%) in KOG, 9,419 (21.33%) in KEGG, 14,762 (33.43%) in Pfam and 11,501 (26.05%) in Swiss-Prot.

For *L*. *simplicicornis* 17,478 unigenes (39.47%) were found in at least one database, 16,928 (38.23%) in NR, 9,415 (21.26%) in GO, 4,978 (11.24%) in COG, 10,973 (24.78%) in KOG, 5,999 (13.55%) in KEGG, 11,441 (25.84%) in Pfam and 9,369 (21.16%) in Swiss-Prot.

**Fig 3:**
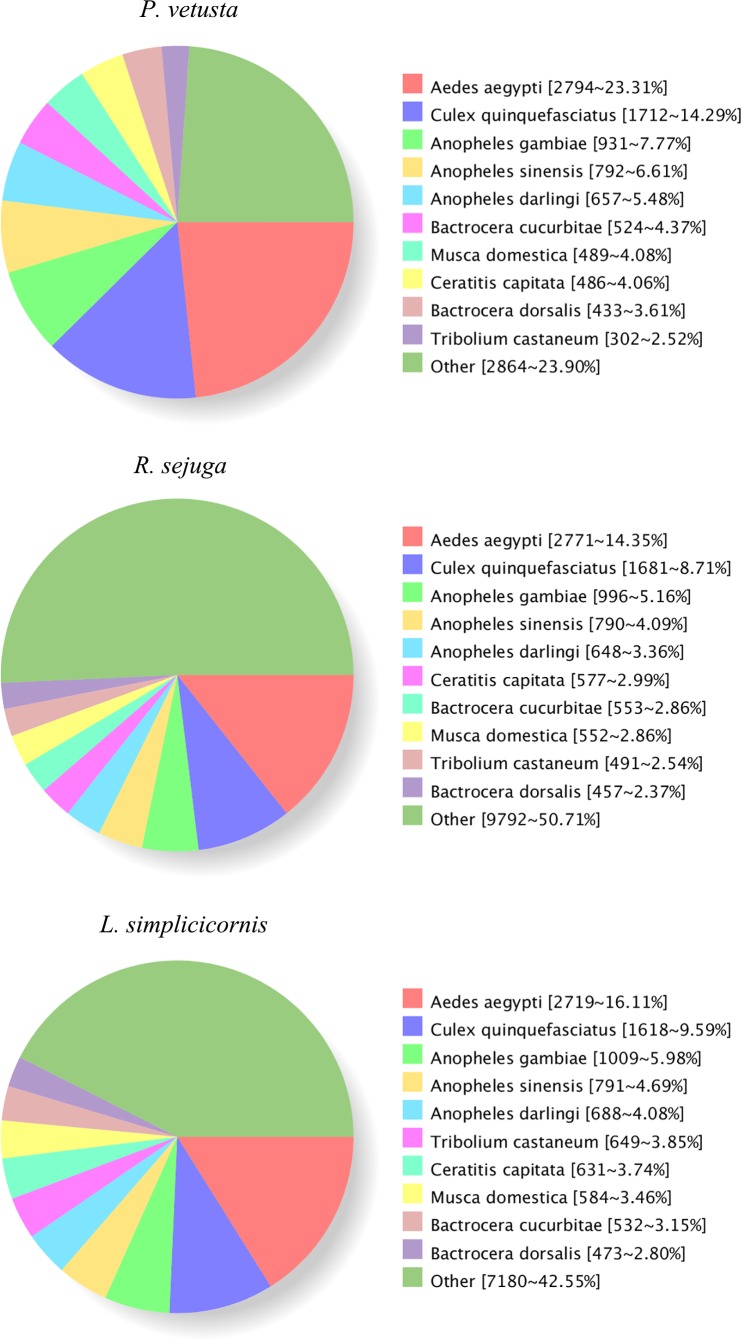
Results summary for sequence-homology search against NCBI NR database.

After searching against the NR database, we obtained the sequence-homology distributions for each species (Fig 3). The majority of matches were with known genes from *Aedes aegypti*, followed by *Culex quinquefasciatus*, *Anopheles gambiae*, *Anopheles sinensis* and *Anopheles darlingi*.

### Classification of unigenes

**Fig 4:**
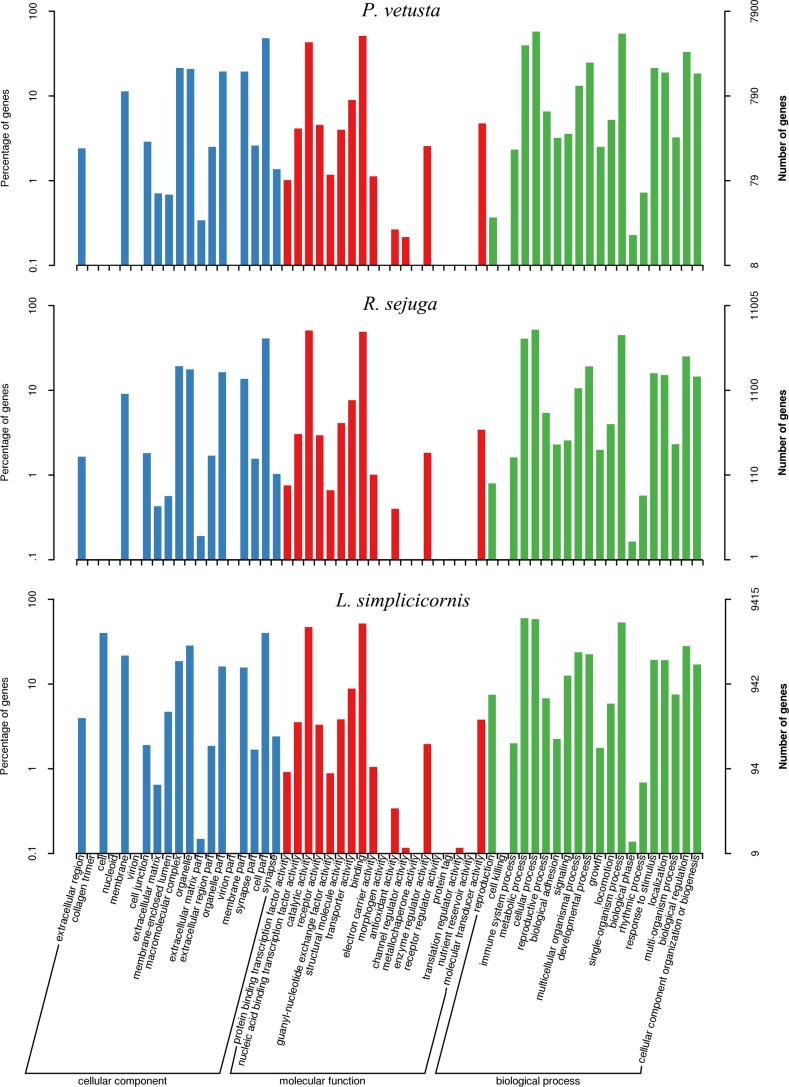
Gene ontology (GO) assignments for the three species. Results are summarized under three main GO categories: biological process, cellular component and molecular function. The left Y-axis represents the percentage of a specific category of genes in each main category. The right Y-axis represents the number of genes in the same category.

For GO analysis, unigenes of each species were divided into three categories: cellular component (CC), molecular functions (MF) and biological processes (BP) [44] (Fig 4). In the CC category, the most abundant terms annotated to the unigenes in both *P*. *vetusta* and *R*. *sejuga* were ‘cell part’ (3,786 and 4,515), ‘macromolecular complex’ (1,692 and 2,125) and ‘organelle’ (1,641 and 1,936), whereas in *L*. *simplicicornis*, the most abundant terms were ‘cell’ (3,778), ‘cell part’ (3,778) and ‘organelle’ (2,680). The MF category mainly comprised proteins involved in ‘binding’ (4,037, 5,410 and 4,886), ‘catalytic activity’ (3,396, 5,571 and 4,431) and ‘transporter activity’ (709, 841 and 833) for all three species. For the BP category, the mostly highly represented terms in all three species were ‘cellular process’ (4,555, 5,699 and 5,474), ‘single-organism process’ (4,286, 4,942 and 5,005) and ‘metabolic process’ (3,132, 4,472 and 5,630). A summary of GO term assignment is presented in S5 Table.

**Fig 5:**
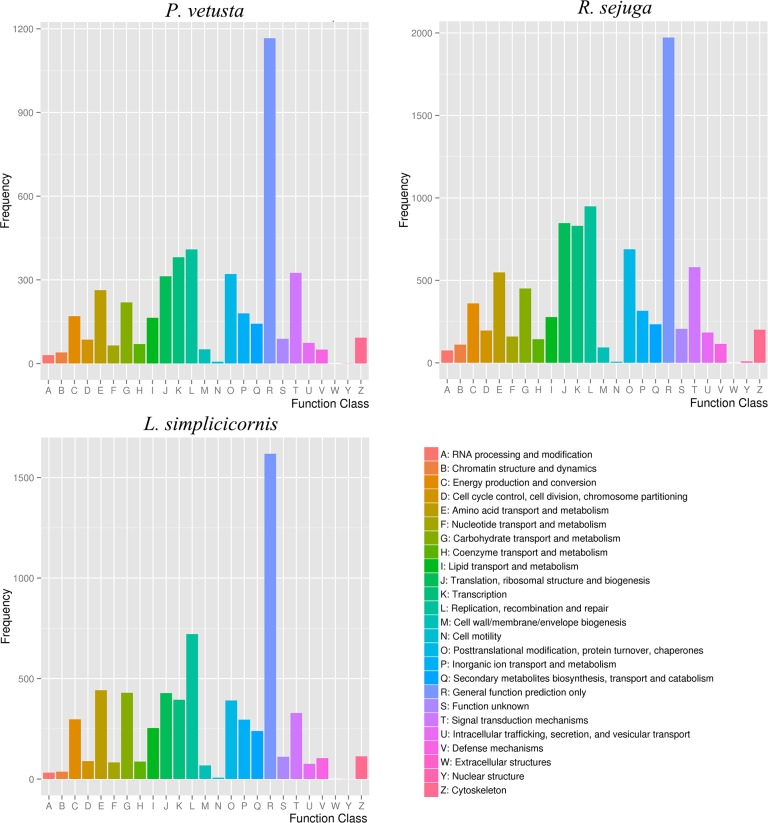
COG functional classification for the three species. Unigenes of each species with significant homologies in the COG database were classified into 25 COG categories.

**Fig 6:**
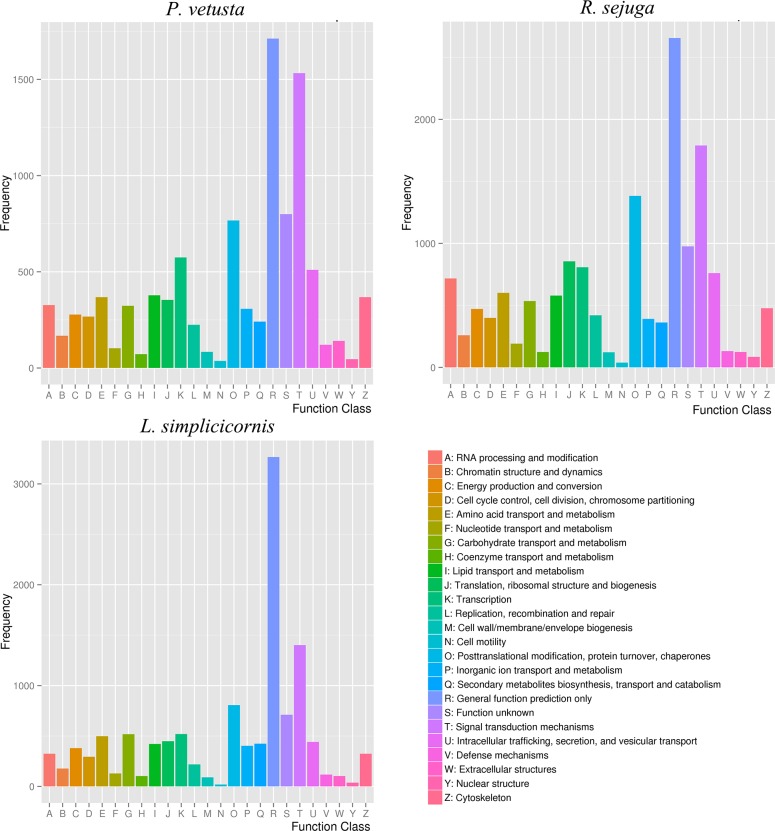
KOG functional classification for the three species. Unigenes of each species with significant homologies in the KOG database were classified into 25 KOG categories.

Unigenes of each species were assigned to COG (Fig 5) and KOG (Fig 6) classification and divided into 25 specific categories. For COG analysis, the largest two groups were the ‘general functional prediction only’ (1,166, 1,972 and 1,618) and ‘replication, recombination and repair’ (409, 949 and 721). The next largest groups in *P*. *vetusta* were ‘transcription’ (381), ‘signal transduction mechanisms’ (325), ‘posttranslational modification, protein turnover and chaperones’ (321) and ‘translation, ribosomal structure and biogenesis’ (313), whereas in *R*. *sejuga*, these groups were also the next most common groups but in a different order: ‘translation, ribosomal structure and biogenesis’ (847), ‘transcription’ (831), ‘posttranslational modification, protein turnover and chaperones’ (689) and ‘signal transduction mechanisms’ (580). Differing from the two species, the third through sixth most abundant groups in *L*. *simplicicornis* were ‘amino acid transport and metabolism’ (442), ‘carbohydrate transport and metabolism’ (429), ‘translation, ribosomal structure and biogenesis’ (428) and ‘transcription’ (395). For KOG analysis, the largest two groups were the ‘general functional prediction only’ (1,712, 2,656 and 3,266) and ‘signal transduction mechanisms’ (1,533, 1,790 and 1,401). However, the middle frequency functional groups were quite different between the three species. For both COG and KOG analysis, only a few unigenes in each species were assigned to ‘cell motility’ and ‘nuclear structure’. A summary of COG and KOG functional classifications is presented in S6 and S7 Tables.

We also mapped the unigenes from each species onto the KEGG database to identify biochemical pathways [45]. In total, for *P*. *vetusta*, *R*. *sejuga* and *L*. *simplicicornis* respectively 6,091, 9,419 and 5,999 unigenes were assigned to 258, 261 and 208 KEGG pathways. A summary of the KEGG pathways annotated is presented in S8–S10 Tables. The largest categories of biochemical-related unigenes in *P*. *vetusta* were those associated with purine metabolism (151; 2.48%), followed by unigenes that were involved in the PI3K-Akt signaling pathway (137; 2.25%), rap1 signaling pathway (135; 2.22%), focal adhesion (135; 2.22%), MAPK signaling pathway (131; 2.15%), and protein processing in endoplasmic reticulum (131; 2.15%), whereas in *R*. *sejuga*, the largest categories were those associated with ribosome (308; 3.27%), followed by unigenes that were involved in purine metabolism (295; 3.13%), spliceosome (292; 3.10%), RNA transport (262; 2.78%), protein processing in endoplasmic reticulum (247; 2.62%), and pyrimidine metabolism (199; 2.11%) and for *L*. *simplicicornis*, the largest categories were those associated with ribosome (188; 3.13%), followed by unigenes that were involved in protein processing in endoplasmic reticulum (139; 2.32%), purine metabolism (133; 2.22%), RNA transport (126; 2.10%), carbon metabolism (124; 2.07%), and spliceosome (108; 1.80%).

### Phylogenetic analysis

**Fig 7:**
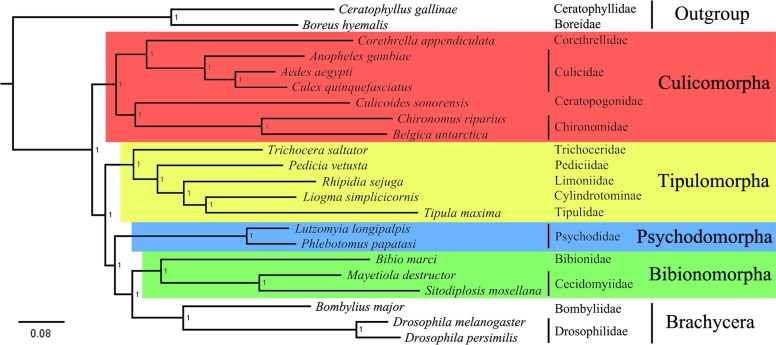
Phylogenetic tree. The tree was inferred through a maximum-likelihood analysis of amino acid sequence data of 1,709 single-cope orthologs genes. Branch lengths correspond to the number of changes on that branch. Numbers adjacent to each node are BV.

**Fig 8:**
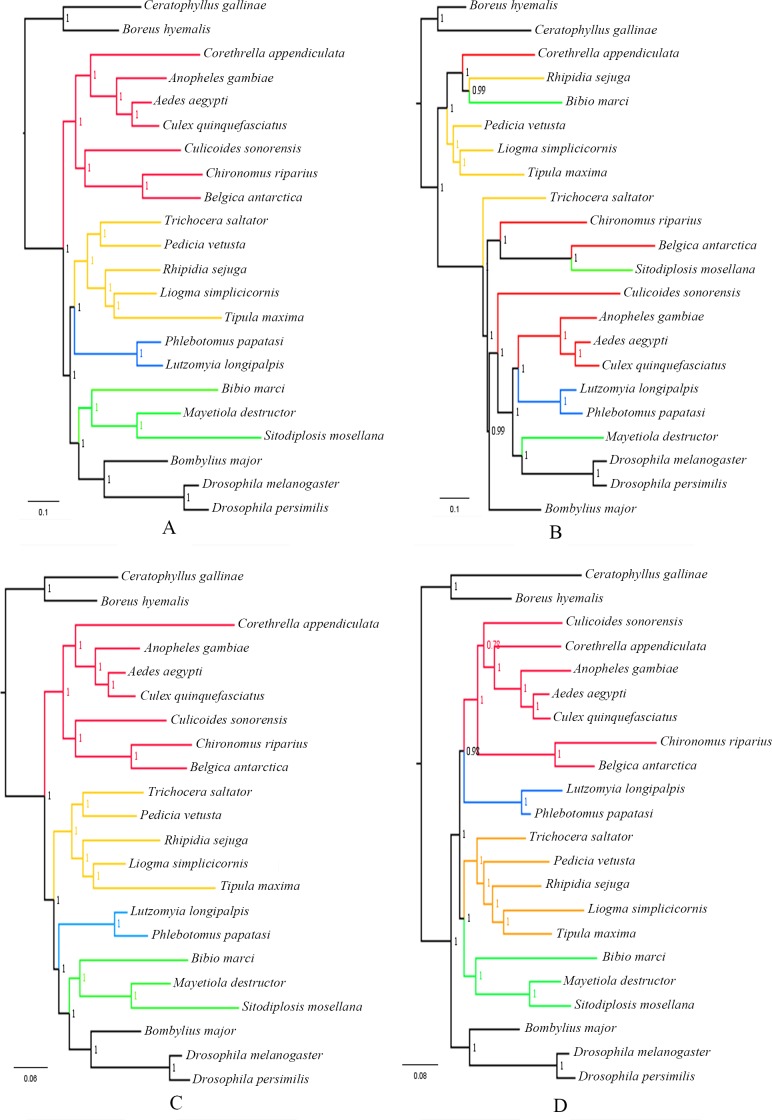
Phylogenetic tree. The tree was inferred through a maximum-likelihood analysis of amino acid sequence data of **(A)** 73 genes involved in ATP binding. **(B)** 13 genes involved in receptor activity. **(C)** 43 genes involved in the biological process associated with wing. **(D)** 7 genes involved in the biological process associated with olfactory. Branch lengths correspond to the number of changes on that branch. Numbers adjacent to each node are BV.

A phylogenetic tree, which included representatives of all tipulomorph families plus representatives of other lower dipteran families, was constructed based on the 1,709 single-copy orthologs genes using the ML method (Fig 7). In addition, according to the annotations of these single-copy orthologous genes, the following four groups of genes were also selected to construct ML trees (Fig 8) to make comparisons and provide insights into the phylogenetic importance of the groups of genes: 73 genes involved in ATP binding, 13 genes with receptor activity, 43 genes from the ‘biological process associated with wing’ group and 7 genes from the ‘biological process associated with olfaction’ group (S11 Table). In the tree based on all 1709 single-copy genes (Fig 7), the monophyly of each infraorder was strongly supported. This result was also strongly supported in three of the four analyses based on GO term group; the exception was the ‘receptor activity’ analyses in which Psychodomorpha is the only monophyletic infraorder (Fig 8B). ‘Nematocera’, or the ‘lower’ Diptera, was, as expected, paraphyletic. Relationships among the four major infraorders of lower Diptera in the 1709-gene analysis was Culicomorpha + (Tipulomorpha + (Psychodomorpha + (Bibionomorpha + Brachycera))). Of the GO group trees, the ‘biological process associated with wing’ analysis (Fig 8C) had the same infraorder topology as the 1709-gene tree, ‘ATP binding’ analysis (Fig 8A) supported a sister group relationship between Tipulomorpha and Psychodomorpha, and the ‘biological process associated with olfaction’ analysis (Fig 8D) supported nematoceran monophyly, with Tipulomorpha as sister to Bibionomorpha. Infraordinal relationships could not be inferred from the ‘receptor activity’ analysis, as the monophyly of 3 of the 4 infraorders was not supported. The 1709-gene analysis (Fig 7) and one of the four GO-group (Fig 8D) supported the traditional concept of Tipulomorpha as containing Trichoceridae separate from Tipuloidea; in two of the other GO-group analyses Trichoceridae was nested within Tipuloidea (Fig 8A and 8C). As to the interfamilial relationships in Tipuloidea, Pediciidae was sister-group to a clade containing Limoniidae + (Cylindrotomidae + Tipulidae) in both the 1709-gene analysis and one of the GO-group analyses, whereas Pediciidae + Trichoceridae was sister to the remaining tipuloid families in the other two GO-group analyses (Fig 8A and 8C). Nodal support was uniformly strong across the analyses.

### FcLM analysis

**Fig 9:**
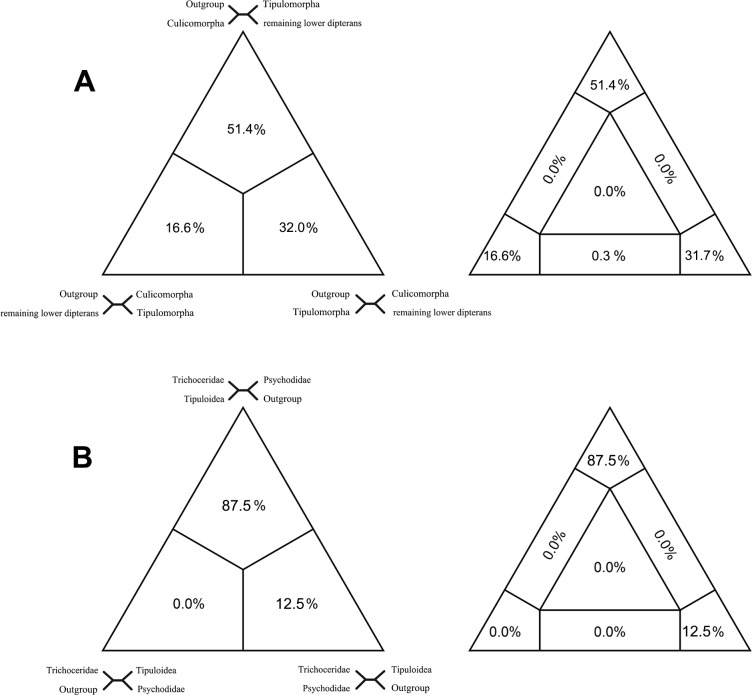
Results of Four-cluster Likelihood Mapping as 2D simplex graphs. (A) Question 1. (B) Question 2.

To evaluate signal for alternative resolutions of the Diptera tree we used Four Cluster Likelihood Mapping (FcLM) [37] to compare support for two major questions: (1) which infraorder is placed basally as sister to all remaining Diptera in our study? And (2) does our data support Trichoceridae placed in the Tipulomorpha (Table 3)? FcLM analysis favored Culicomorpha (51.4%) over either Tipulomorpha (32.0%) or a clade comprising of Culicomorpha + Tipulomorpha (16.6%) (Fig 9A). Quartet mapping also showed strong support for Trichoceridae + Tipuloidea (Tipulomorpha monophyly) (87.5%) over either a sister grouping between Trichoceridae and Psychodidae (12.5%) or Trichoceridae outside of Tipuloidea + Psychodidae (0.0%) (Fig 9B). Both the results are compatible with the results of the ML tree based on all 1709 single-copy genes.

**Table 3:** The three datasets designed to address three phylogenetic questions.

Phylogenetic question	Groups	Included insect families (if more than one)	Number of species
1. Which infraorder is the sister-group of the remaining Diptera?	G1: Outgroup	Boreidae, Ceratophyllidae	2
	G2: Culicomorpha	Culicidae, Chironomidae, Ceratopogonidae, Corethrellidae	7
	G3: Tipulomorpha	Trichoceridae, Pediciidae, Limoniidae, Cylindrotominae, Tipulidae	5
	G4: remaining lower dipterans	Psychodidae, Bibionidae, Cecidomyiidae	5
2. Is Trichoceridae part of Tipulomorpha?	G1: Trichoceridae		1
	G2: Tipuloidea	Pediciidae, Limoniidae, Cylindrotominae, Tipulidae	4
	G3: Psychodidae		2
	G4: Outgroup	Boreidae, Ceratophyllidae	2

## Discussion

Recent studies have demonstrated that whole transcriptomes can accurately resolve the phylogenetic relationships at various levels within insects [29–30]. A robust phylogeny of Tipulomorpha was obtained using three newly sequenced tipuloidean transcriptomes as well as some published transcriptome data. Strong support values were recovered for both traditionally recognized dipteran phylogenetic relationships as well as some recent novel findings (Fig 7). Several long standing issues in tipulomorph phylogeny are resolved, especially regarding its monophyly and placement with respect to other lower dipteran groups. As for the four major infraorders of lower Diptera, Culicomorpha was the basal branch in our analysis, which was consistent with former molecular [10] and morphological analysis [18]. The placement of Tipulomorpha towards the middle of the nematoceran grade was recovered, with Culicomorpha + (Tipulomorpha + (Psychodomorpha + (Bibionomorpha + Brachycera))). This is consistent with the result using Bayesian analysis from Bertone *et al*. [10].

The monophyly of Tipulomorpha as well as the sister-group relationship between Trichoceridae and Tipuloidea are confirmed. Several morphological characters of both larvae and adults support this sister group relationship, i.e. vein R_2_ ending in R_1_, vein A_2_ reaching the wing margin, reduction of male cerci, development of male terminalia from both imaginal discs and pupal ectoderm, and female cerci with a single article [4–9]. Despite differing in their resolution of the infraorders, both of Bertone *et al*. analyses supported a sister-group relationship between Trichoceridae and Tipuloidea based on multiple nuclear genes [10]. Analysis of whole mitochondrial genome data by Beckenbach did not support a monophyletic Tipulomorpha as Trichoceridae was sister to all other Diptera, however the exclusion of higher variability major genes and codon positions did support a sister-group relationship between Trichoceridae and Tipuloidea [17].

The superfamily Tipuloidea, which has a large number of species, are difficult to identify and really know. Therefore, the choice of taxa could influence the tree due to unpredictable rate dynamics (causing homoplasy or phylogenetic error). However, the family Tipulidae was the sole representative of Tipuloidea included in some previous studies (e.g. [17–18]) potentially biasing those phylogenetic findings. Limited taxon sampling could explain the conflicting results found in most previous studies for the placement and composition of Tipulomorpha. Our use of a very large gene sample may overcome the unpredictable rate effects that can come from insufficient taxon coverage, but additional studies with much broader taxon coverage would be required to fully explore this issue.

Regarding interfamilial relationships in Tipuloidea, our results corroborated Pediciidae as the sister-group to the remaining Tipuloidea, which was consistent with the results proposed by Ribeiro based on an analysis of 88 morphological characters [27] and Petersen *et al*. based on both morphological characters (adult, larvae and pupae) and nuclear sequence data (28S rDNA and CAD) [28]. Petersen *et al*. also showed a sister-grouping of Cylindrotomidae and Tipulidae, however, their placement within the Tipuloidea was less certain as there was no support for the monophyly of Limoniidae [28]. Although Limoniidae was found to be the sister-group of Cylindrotomidae + Tipulidae in the present study, due to the limited number of exemplars sequenced (one per family) we can't rule out the possibility of a paraphyletic Limoniidae as found by Petersen *et al*. [28] with only some members of the family making up the sister-group of Cylindrotomidae + Tipulidae. More research into the higher-level classification of the Limoniidae based on a larger range of representative taxa should be used for phylogenetic analysis in the future.

Analysis of smaller numbers of genes corresponding to specific functional classes were less effective at resolving expected relationships among Tipulomorphan clades. GO clasess are broadly defined and specific aspects of molecular evolutionary rate and both identification or identity as an ortholog would affect both the use of the gene as phylogenetic marker and its ability to resolve a specific question. In general, it seems that larger gene samples that include multiple classes of genes and genes of differing phylogenetic utility are preferable for resolving relationships among the oldest and most diverse fly groups.

## Supporting information

S1 Table Species for which new transcriptome data were generated, with collecting and preservation information. (XLSX)

S2 Table All species included in this study, including previously published data. (XLSX)

S3 Table List of 1,709 ortholog groups included in the ortholog reference set. (XLSX)

S4 Table Reference species used in the orthology reference set. (XLSX)

S5 Table Summary of GO term assignment for unigenes of *P*. *vetusta*, *R*. *sejuga* and *L*. *simplicicornis*. (XLSX)

S6 Table Summary of COG functional classification for unigenes of *P*. *vetusta*, *R*. *sejuga* and *L*. *simplicicornis*. (XLSX)

S7 Table Summary of KOG functional classification for unigenes of *P*. *vetusta*, *R*. *sejuga* and *L*. *simplicicornis*. (XLSX)

S8 Table Summary of the KEGG pathways for unigenes of *P*. *vetusta*. (XLSX)

S9 Table Summary of the KEGG pathways for unigenes of *R*. *sejuga*. (XLSX)

S10 Table Summary of the KEGG pathways for unigenes of *L*. *simplicicornis*. (XLSX)

S11 Table List of the four groups of genes selected to construct ML trees. (XLSX)
